# University students' metaverse attachment and its predictors: escaping from reality

**DOI:** 10.3389/fpsyg.2025.1594256

**Published:** 2025-09-23

**Authors:** Yan Xu, Linmin Chen

**Affiliations:** ^1^School of Transportation, Fujian University of Technology, Fuzhou, China; ^2^School of Marxism, Xi'an Jiaotong University, Xi'an, China

**Keywords:** escape theory, metaverse, virtual place attachment, ELM, contextual fluidity

## Abstract

The most ambitious vision of metaverse technology is to create virtual spaces that offer possibilities equivalent to those in the real world. However, like any emerging technology, the metaverse has sparked controversies and raised questions. Why do individuals seek to migrate to the metaverse? Virtual reality experiences enable people to immerse themselves in virtual environments for hours, interacting with content within these worlds, thus forming another virtual universe—the metaverse—that provides a sense of place and alternative reality. As an emerging research domain, the metaverse holds great potential in offering an alternative habitat for individuals. While the significance of escapism in the metaverse has been discussed in existing literature, what drives university students to escape into the metaverse and its consequences remain unclear. In this study, we leverage Escape Theory to examine how various psychological challenges drive university students to escape reality through the metaverse and develop an attachment to this virtual place. We collected 585 responses from university students who are users of metaverse applications based on virtual reality. The data were analyzed using the Partial Least Squares Structural Equation Modeling (PLS-SEM) technique. Our findings reveal that autonomy problems, study competence problems, relatedness problems, amount of time spent in the metaverse, and interactivity all contribute to university students engaging with the metaverse as a means of escaping reality, subsequently leading to virtual place attachment. This study contributes to metaverse literature by exploring the real-life challenges that may lead university students to engage in metaverse escapism. These results provide a deeper understanding of individuals' perceptions of the metaverse and how the connections between virtual and real spaces translate into attitudes toward metaverse technologies.

## 1 Introduction

The advent of virtual worlds and metaverse platforms has ushered in a new era of digital experiences, transcending physical boundaries and redefining how we interact with technology (Bansal and Axelton, 2023; Chakraborty et al., 2023b; Polisetty et al., 2024). Advances in technology have transformed the ways we connect with those around us (Ashaduzzaman et al., 2022; Jebarajakirthy et al., 2021; Kumari and Kumar, 2023). With the rise of the internet and the widespread adoption of social media applications, virtual worlds have experienced significant growth (Jebarajakirthy and Shankar, 2021; Kumari and Kumar, 2023; MacCallum and Parsons, 2019; Shankar et al., 2022).

The metaverse, often defined as a new era of the internet, integrates virtual reality (VR) devices, interactive avatars, augmented reality (AR) technology, and blockchain technology within a 3D virtual environment (Dwivedi et al., 2022). These technologies enable users to fully enjoy the unparalleled interactivity and immersive experiences offered by the metaverse (Akour et al., 2022; Kumar et al., 2023). The metaverse allows individuals to spend extended periods in immersive virtual environments, interacting with content that provides both a refuge from reality and an illusion of an alternative world.

While escapism has long been associated with gaming, social media, and virtual reality, the metaverse introduces a qualitatively different set of affordances. Unlike traditional gaming, which is goal-directed and episodic, the metaverse is a persistent, identity-rich, and socially co-constructed environment. Compared to VR—which often provides isolated, task-specific simulations—the metaverse enables users to maintain continuous digital identities, engage in real-time economic and social exchanges, and traverse across diverse functional spaces including education, work, entertainment, and commerce. This multifunctionality and interoperability distinguish the metaverse as a unique medium for psychological displacement.

These technological and functional distinctions significantly reshape the psychological dynamics of escapism. The immersive nature of the metaverse intensifies users' sense of spatial and emotional detachment from the real world; its persistence reinforces continuity of escapist engagement across time; and its multi-functionality enables users to substitute real-world roles with virtual ones—such as student, worker, or friend—within the same environment. Consequently, escapism in the metaverse is not merely a temporary retreat, but a potentially habitual relocation of self-experience, making it qualitatively different from escapism in gaming or social media. This raises new theoretical questions about long-term psychological displacement and digital identity anchoring.

Technological advancements have significantly altered social interactions and the ways university students engage with one another (Ashaduzzaman et al., 2022; Gupta et al., 2024). Previous research has acknowledged that our current fast-paced lifestyles contribute to emerging mental health challenges, reduced physical activity, and increased prevalence of obesity and other health issues—all of which can generate a desire for escape (Thoits, 2010).

Given this context, it is unsurprising that researchers are increasingly advocating for comprehensive studies on various aspects of metaverse technology. However, research on the metaverse remains in its infancy (Chakraborty et al., 2023a; Kim et al., 2023; Park and Lim, 2023). Current investigations predominantly focus on conceptual studies (Allam et al., 2022; Barrera and Shah, 2023; Dwivedi et al., 2022; Kim, 2021; Tlili et al., 2022) or applications of machine learning techniques (Almarzouqi et al., 2022), with few empirical studies exploring the behaviors of university students in the metaverse.

As the global adoption of immersive technologies accelerates, the metaverse is rapidly transforming from a speculative concept into an integral part of digital life (Dwivedi et al., 2022). Particularly among university students, this shift is evident in the widespread use of platforms like Roblox, Zepeto, and Genshin Impact, which now function not only as entertainment tools but as social and emotional spaces (Zimmer, 2022; Oh et al., 2023). These developments underscore the urgent need to empirically examine why students are turning to the metaverse—not just for interaction, but as an alternative psychological refuge from real-world stressors.

Online social networking is highly significant for university students as it facilitates their developmental tasks during early adulthood, including establishing a sense of identity and forming meaningful relationships with others. It is also frequently utilized for educational purposes (Shen et al., 2020). Compared to their experiences during high school, university students face fewer barriers when accessing the internet and experience less control from others regarding its use. Consequently, they increasingly frequent the metaverse (Zimmer, 2022).

What drives university students to leave the real world and escape into virtual spaces like the metaverse? While escaping to the virtual world is not equivalent to physical escape, it can be compared to it because it involves minimal cost and is easily reversible (Coulson et al., 2020). This type of escape is characterized by its temporary nature, frequent transitions, and low migration costs, significantly replacing the traditional unidirectional escape patterns (Salamońska and Czeranowska, 2021).

Escape is regarded as one of the primary coping mechanisms for dealing with everyday adversity, stress, and tension (Kosa and Uysal, 2020). Since the 1950s, escapism has been recognized as a crucial determinant in explaining motivations for consuming media content, serving as a strategy to avoid the psychological discomfort experienced in the “real” world (Han et al., 2022). In this context, addiction to social media and online gaming, as a specific form of escapism, has been rapidly increasing, raising concerns about its impact on societal wellbeing (Andreassen et al., 2016).

This study aims to address the research gap by focusing on university students' willingness to escape to the metaverse and shift a significant portion of their daily activities to virtual worlds, as well as the predictors of this behavior. We hypothesize that greater acceptance of virtual world development and willingness to use it are associated with previous positive experiences in virtual spaces, primarily stemming from attachment to currently used virtual environments and the perception that these spaces better meet their needs than real-world environments (Srivastava and Chandra, 2018). Furthermore, we anticipate that, like any new and evolving technology, the development of the metaverse will raise certain concerns and issues.

This study aims to address the following research questions: What are the risks and potentials associated with university students escaping from reality to virtual reality, and how should virtual reality be studied and developed to positively promote their mental wellbeing? We must prepare for the similar or even more severe impacts of reality escape through virtual reality experiences, as it is currently the most prominent technology with the potential to immerse users completely in virtual, computer-generated environments. As research and use cases for multi-sensory virtual reality continue to grow, the potential mental health impacts of this technology, along with the resulting ethical issues, have become critical topics in virtual reality research. In this paper, we emphasize the necessity of designing virtual reality escape experiences centered around university students to contribute positively to society. Based on these considerations, we propose future research directions for virtual reality experiences, encompassing both conceptual and empirical studies. By doing so, we aim to contribute to the development of future educational research and enhance the understanding of virtual reality's role in addressing psychological and societal challenges.

Despite growing academic interest in escapism, prior research has primarily explored this phenomenon in the contexts of online gaming and social media (Chang et al., 2018), with limited attention paid to its manifestation in immersive metaverse environments. This represents a critical gap, especially considering that university students are among the earliest and most frequent users of metaverse platforms. Unlike casual users, university students face a unique set of developmental pressures—including autonomy challenges, academic stress, and social isolation—which have been empirically linked to increased digital escapism and metaverse engagement (Pal and Arpnikanondt, 2024). Recent empirical studies have confirmed that young adults with high psychological stress or unmet needs are more likely to engage in digital escapism through virtual platforms (Marques et al., 2023; Pal and Arpnikanondt, 2024). For instance, Pal and Arpnikanondt (2024) demonstrated that students facing anxiety or relatedness deficits tend to form stronger emotional bonds with metaverse spaces. The metaverse offers not just a digital space but an immersive psychological outlet that could potentially influence students' emotional regulation and identity development (Ud Din and Almogren, 2023).

Given this context, the urgency of studying escapism among university students in the metaverse lies in both theoretical and practical considerations. Theoretically, it expands current escapism models to account for immersive and interactive environments. Practically, it addresses concerns about digital detachment and long-term psychological effects among a vulnerable population.

Based on this motivation, the objectives of the study are:

To investigate the internal psychological challenges (autonomy, study competence, and relatedness problems) that lead university students to escape into the metaverse;

To examine how external features of the metaverse (time spent, interactivity, visual attractiveness) influence attitudes toward escapism;

To explore how escapism predicts virtual place attachment.

## 2 Literature review

### 2.1 Escape theory

Escape theory posits that when individuals perceive a problem they cannot solve through their own capabilities, and there exists an alternative task that they can immediately engage in, this task becomes a means of escape (Kurzban et al., 2013). Escaping reality refers to the need for individuals to “leave” their real-world lives cognitively and emotionally (Vaisey and Kiley, 2021). Escapism is often characterized by content that is pleasurable, such as the experiences offered by (online) games, which elicit euphoria and help individuals divert their attention away from stressful stimuli or situations.

According to escape theory, online information and communication technologies can, to some extent, help individuals subjectively escape from negative life circumstances and cope with the daily pressures of modern society (Roach and Simpson, 2016). Escape motivation refers to the tendency of individuals to utilize virtual environments to avoid negative emotions, stress, and problems associated with the real world (Pal and Arpnikanondt, 2024). This aligns closely with escape theory, which asserts that when individuals feel unable to cope with psychological discomfort, they develop an escape-oriented mindset, such as retreating into the online world (Maynard, 2019).

Escape theory highlights the psychological motivations driving users toward the metaverse, a relationship recently validated by empirical studies linking escapism with virtual place attachment and anxiety regulation (Orazi et al., 2023; Pal and Arpnikanondt, 2024). Individuals use virtual worlds to realize fantasies, establish connections with others, and compensate for deficiencies in real-world social settings (Pal and Arpnikanondt, 2024). Problems and frustrations in the real world often stem from unmet psychological needs, which are directly related to personal wellbeing (Deci and Ryan, 1985). Escape theory suggests that when individuals face threats in one environment (e.g., the real world), they may initiate a self-affirmation process to regain a sense of capability (Sherman and Cohen, 2006). In doing so, they may seek solace in another environment, such as the metaverse. This phenomenon mirrors the concept of escaping reality and validates the inclusion of this construct within our research model.

These psychological needs for escaping reality drive university students to explore the diverse features, functions, and services offered by virtual worlds. Such activities further enhance their immersion in and attachment to the virtual world, allowing them to psychologically distance themselves from real-world problems and deepen their attachment to the metaverse. This phenomenon has been observed across various contexts, including live-streaming services (Peng et al., 2024), brand perception (Japutra et al., 2018), and tourism-based services (Kim et al., 2020).

Thus, university students' attachment to the metaverse and their frequent retreats to virtual worlds underscore the need to study the drivers of escapist behaviors toward the metaverse. As the quality of virtual experiences continues to improve, it is crucial to refocus on psychological and social issues to minimize the potential negative impacts of current and future virtual reality technologies.

Building on these theoretical foundations, Orazi et al. (2023) developed and validated a multidimensional scale of consumer escapism, providing empirical tools to measure individuals' motivations for escaping reality via digital platforms. Pal and Arpnikanondt (2024) further applied this framework to the metaverse context, demonstrating that higher levels of anxiety are linked to increased virtual attachment, mediated by escapism tendencies. These findings strengthen escape theory's validity and guide our variable selection.

In this study, we define escaping to the metaverse as a form of digital escapism involving voluntary psychological disengagement from the real world and immersion into virtual environments, driven by emotional, social, or competence-related pressures (Orazi et al., 2023). Unlike casual usage, this behavior is goal-directed and often recurrent, serving as a coping strategy for unmet psychological needs.

### 2.2 Elaboration likelihood model

The Elaboration Likelihood Model (Petty and Cacioppo, 2012) distinguishes between two routes of persuasion: the central route, which requires deep cognitive processing, and the peripheral route, which relies on surface-level cues. In the context of the metaverse, internal psychological states—such as autonomy problems, study competence frustration, and relatedness deficits—align with central route processing, as they represent high-effort, personally relevant factors that shape users' motivation to escape. Conversely, environmental features such as interactivity, visual attractiveness, and time spent function as peripheral cues, influencing users' escape behavior with minimal cognitive elaboration (Jayawardena et al., 2023). This classification enables us to map internal and external constructs directly onto ELM, enhancing conceptual clarity and model integrity.

ELM has been widely studied in various contexts as a persuasion model for attitude change, including online gaming (Wang et al., 2019), social media influencers (Teng et al., 2014), virtual reality advertisements in the hotel industry (Leung et al., 2020), mobile shopping (Chen et al., 2018), live streaming to boost product sales (Chen et al., 2023), augmented reality (AR) and virtual reality (VR) video ads (Jayawardena et al., 2023), IT adoption (Lee, 2012), and mobile health applications (Chen et al., 2023). While metaverse attachment is a relatively new concept, it has only recently started to gain research attention. Given that ELM has been extensively used in technology studies, particularly in AR and VR contexts, this study extends its conceptual model based on ELM (Abbasi et al., 2023; Park and Yoo, 2020).

ELM is instrumental in exploring the cognitive processes that influence user behavior, particularly its central role in cognition and adaptability to virtual environments. This study aims to leverage ELM to gain critical insights into the decision-making and thought processes unique to the metaverse, thereby enhancing understanding in this field.

In the domain of immersive technologies, several studies have applied the ELM to examine how sensory and peripheral cues influence user decisions in virtual environments (Jayawardena et al., 2023). For instance, Jayawardena et al. (2023) found that visual and interactive cues in VR/AR advertisements significantly shape user attitudes through the peripheral route of the ELM. In the context of mobile commerce and digital platforms, researchers have confirmed that emotionally engaging elements enhance user attention and subsequent attitude change (Ahmed et al., 2024). These findings affirm the suitability of ELM as a theoretical framework to explain both central (e.g., autonomy, competence) and peripheral (e.g., visual, interactivity) influences in metaverse contexts.

To understand the intentions behind university students' attachment to the metaverse after escaping reality, this study adopts ELM as its theoretical framework. Based on the metaverse attachment literature, this research identifies autonomy problems, study competence problems, and relatedness problems as internal factors that contribute to a broader attachment to the metaverse. On the other hand, external factors often involve multiple cues that influence subsequent actions, such as engaging design elements, source credibility, and production style (Teng et al., 2014). Accordingly, this study selects amount of time spent, interactivity, and perceived visual attractiveness as external routes.

An individual's attitude can shift depending on the sufficiency of external information received (Erkli, 2022), and such attitudes are often expressed as intentions to act (Ajzen and Fishbein, 1977). Thus, external cues can influence users' attitudes toward the metaverse, which in turn affect their escapist behaviors in this virtual space. By integrating ELM, this study aims to provide a comprehensive framework for understanding the cognitive and behavioral drivers of metaverse attachment, contributing to the broader discourse on virtual environments and their impact on user behavior.

This study adopts Escape Theory to explain why users seek the metaverse—due to psychological strain—and ELM to explain how immersive features trigger escapist engagement via central or peripheral routes. Internal factors (e.g., autonomy frustration) follow the central route, while external cues (e.g., interactivity, aesthetics) activate peripheral processing. This integrated lens links personal motivation with persuasive digital design.

### 2.3 Theoretical integration: SDT and escape theory

This study adopts a dual-theoretical approach by integrating Self-Determination Theory (SDT) and Escape Theory to explain the motivational antecedents and behavioral outcomes of digital escapism in the metaverse. SDT provides a foundational account of psychological strain: when individuals experience unmet basic psychological needs—autonomy, competence, and relatedness—they develop emotional states such as anxiety, helplessness, or alienation (Ryan and Deci, 2000). Emotional strain serves as a mediator linking need frustration (SDT) and escapist behavior (Escape Theory). These emotional states form the basis of psychological distress.

Escape Theory complements this by explaining how individuals respond to such distress. It posits that people under strain seek to withdraw from self-awareness or reality by engaging in alternate environments (Baumeister, 1991). In immersive contexts such as the metaverse, this coping manifests as escapist engagement, where users immerse themselves in virtual environments to alleviate discomfort.

This integration thus creates a sequential explanatory framework: SDT accounts for the emergence of internal pressure, while Escape Theory explains how this pressure leads to behavior (e.g., metaverse use) and emotional outcomes (e.g., virtual place attachment). Together, the two theories illuminate both the why and how of digital escapism, offering a comprehensive understanding of user behavior in immersive digital spaces.

### 2.4 Escaping to the metaverse and virtual place attachment

Virtual place attachment is conceptualized as the affective and behavioral bond individuals develop toward specific digital environments that offer emotional comfort, identity expression, or social belonging (Trabka, 2019; Williams and Vaske, 2003). It involves three components: emotional bonding, place identity, and commitment to virtual participation.

Place attachment is broadly defined as the emotional, cognitive, and behavioral bond that individuals form with a particular place (Lewicka, 2011). In numerous studies, place attachment has been found to enhance an individual's desire to remain in a specific location. Trabka (2019) suggested that place attachment to a new location initially develops based on the attractiveness of the place and the resources it offers. Positive emotions associated with experiences in the new location can make individuals feel comfortable and safe, which, in turn, fosters a desire to continue exploring and enjoying the opportunities the place provides. This process ultimately helps individuals establish a stable and profound connection with the new location.

For university students, the metaverse provides an alternative virtual space that transcends the limitations of reality. In the metaverse, they can engage in social interactions through avatars, participate in immersive activities, and access educational resources. Escape theory suggests that escaping reality may have therapeutic effects by alleviating stress (Giardina et al., 2021). Thus, by escaping reality and entering the metaverse, university students not only experience enjoyment but also reduce the psychological burdens brought by real-world challenges, gradually forming an attachment to the virtual world. Similar to other forms of escapism, such as sports, shopping, tourism, or gambling, university students in the metaverse are often less aware of or entirely disengaged from their surrounding reality (Mogaji et al., 2023).

The ability of the metaverse to simulate realistic interactions holds a unique appeal for university students. Through avatars, they can mimic hand movements, arm gestures, facial expressions, and even eye contact to interact and communicate with others. This experience satisfies social needs that are often unmet in real life, offering them an opportunity to “get away from it all” (Kuo et al., 2016). Such comprehensive engagement and immersive interaction make it easier for university students to develop attachment to the metaverse. Therefore, we hypothesize: H1. Escaping to the metaverse is positively correlated with virtual place attachment.

### 2.5 Internal factors of escaping to the metaverse

Prior studies have largely examined predictors of escapism in fragmented contexts—some focusing on psychological antecedents (e.g., stress, social deficits), others on immersive features (e.g., time, presence). For example, Orazi et al. (2023) validated escape motivation scales in digital use, while Li et al. (2021) linked unmet social needs to mobile phone escapism. In the metaverse context, Pal and Arpnikanondt (2024) found that both internal psychological strain and external design features jointly shape users' escape intention. This study builds on and integrates these strands by categorizing predictors into internal (autonomy, competence, relatedness) and external (time, interactivity, attractiveness) factors.

Escaping reality should provide university students with an effective way to cope with the stress resulting from unmet basic needs in real life. As current research highlights, escapism is highly relevant in the context of the metaverse (Floridi, 2022; Han et al., 2022; Mogaji et al., 2023). However, such propositions remain conceptual and require empirical validation. Internal issues in the real world can be categorized into three types (Deci and Ryan, 1985), with the first being autonomy problems, which refer to the degree of pressure individuals feel when performing tasks under limited free will (Chen et al., 2015). Autonomy problems arise when university students' freedom to make decisions in their studies or daily lives is constrained by others, thereby limiting their sense of autonomy (Liao et al., 2020). In the metaverse, university students are able to freely express their desires and intentions, such as by using or customizing avatars (Liao et al., 2020). On platforms like Roblox, students can even drive their dream cars and experience ultimate in-car comfort based on real-world data (Henz, 2022). Through the creation of digital twins and their use in the metaverse, students can experience a sense of freedom that is unattainable in real life (Voinea et al., 2022). Thus, the limited autonomy in the real world may motivate university students to escape to the virtual world to gain greater freedom.

The second internal problem arises from frustration related to study competence issues. Study competence refers to students' sense of control and efficacy during activities. When students experience a lack of achievement, it can lead to study competence issues, causing them to doubt their abilities (Chen et al., 2015; Martela and Riekki, 2018).

Students who experience low academic efficacy are more likely to seek psychological compensation through virtual platforms, a pattern supported by findings that VR-based learning can rebuild perceived competence and motivation (Castronovo et al., 2024), as part of physical education, can be significantly improved if training sessions are conducted in a virtual reality-based metaverse environment, thereby enhancing teaching quality and the student experience (Huang et al., 2024). Similarly, drivers who failed real-life driving tests and experienced a sense of failure demonstrated improved driving skills and knowledge after participating in a serious gaming platform based on online virtual reality (Likitweerawong and Palee, 2018).

These examples indicate that escaping to the virtual world can help offset the frustration associated with study competence issues in real life. Escapism can, therefore, be considered an intuitive way for university students to cope with challenges stemming from study competence problems in the real world.

Relatedness problems are a significant psychosocial factor driving university students to escape to the metaverse. Relatedness refers to the degree to which individuals establish connections, feel cared for, and integrate into a community (Henz, 2022). When relatedness needs in the real world are unmet, such as difficulties in socializing with peers or estranged intimate relationships, individuals may feel lonely and excluded. These frustrations prompt them to seek alternative social environments, with the metaverse offering such a virtual platform.

The metaverse, through its interactivity and immersive experiences, can simulate and even enhance social support networks. For example, Oh et al. (2023) noted that the metaverse strengthens users' sense of social presence in virtual environments, providing a greater sense of belonging and supportive interactions. This not only alleviates loneliness but also motivates users to continue engaging in the virtual world (Li et al., 2021).

Furthermore, the metaverse addresses students' social needs through virtual campuses, interest groups, and similar social scenarios. These settings create an inclusive environment, compensating for the lack of emotional and social support in the real world. For instance, virtual activities help students overcome social barriers, offering a safe space for interaction without the pressures of real-world expectations (Chen et al., 2015). The metaverse not only temporarily alleviates real-world challenges but also enhances subjective wellbeing through sustained social support networks (Oh et al., 2023).

When university students perceive their real-life relationships as unfulfilling, they are more likely to turn to the metaverse as an alternative social space. Studies have shown that the freedom and enjoyment of social interactions in virtual environments can enhance users' wellbeing (Ranzini and Rosenbaum, 2020). Activities such as multiplayer games or role-playing not only improve social skills but may also positively influence real-world relationships, reducing isolation.

Thus, we hypothesize:

H2. University students' autonomy problems in the real world are positively correlated with their escape to the metaverse.
H3. University students' study competence problems in the real world are positively correlated with their escape to the metaverse.
H4. Relatedness problems in the real world are positively correlated with university students' escape to the metaverse.

### 2.6 External factors of escaping to the metaverse

It is important to distinguish relatedness problems, which stem from users' perceived social disconnection in real life, from interactivity, which refers to system-enabled real-time engagement in the metaverse. While both may influence social satisfaction, relatedness is an internal state, whereas interactivity is an external system characteristic (Jin et al., 2017).

In contrast to internal psychological states, external predictors of escapism reflect affordances of the digital environment that facilitate immersion and behavioral engagement. Drawing from the Elaboration Likelihood Model (Petty and Cacioppo, 2012) and immersive media theory (Rössler, 2017), this study selects time spent in the metaverse, perceived interactivity, and visual attractiveness as key environmental factors that enhance users' psychological distance from reality. Empirical findings suggest that prolonged time spent in immersive virtual worlds increases social presence and psychological detachment (Bojić et al., 2025; Oh et al., 2018a), while high interactivity and aesthetic appeal serve as peripheral cues that promote emotional involvement and sustained engagement (Ahmed et al., 2024; Jayawardena et al., 2023). Together, these elements shape the external environment that encourages escapist behavior in the metaverse.

Brands and companies are encouraged to seek opportunities to increase consumers' time spent (immersive time) and ensure active engagement, attachment, and involvement during their time in the metaverse (Mogaji et al., 2023). Companies and brands must stimulate consumers' immersive time to build their social presence and attachment (Mogaji et al., 2023; Oh et al., 2023).

Based on these arguments, we propose that the amount of time spent by Generation Z consumers positively predicts their social presence and attachment to the metaverse.

The high interactivity of the metaverse provides users with an immersive participatory experience that significantly enhances their emotional and cognitive engagement, allowing for more personalized and meaningful interactions in virtual environments. This immersive interactivity not only boosts user satisfaction and presence (Sundar and Oh, 2019) but also facilitates psychological escapism, enabling university students to temporarily detach from real-life stressors more effectively (Oh et al., 2018b). Furthermore, Kim and Ko (2019) emphasize that interactive elements in virtual worlds serve as a crucial means of alleviating real-world pressures.

As a core demographic of metaverse users, university students face low entry barriers and exhibit high acceptance of virtual environments. Yu (2024) reports that university students view interactivity as a key factor in their metaverse experience. Moreover, user interaction with virtual marketing content has been shown to stimulate a sense of social presence (Yan et al., 2023). Specifically, increased media richness, interactivity, and vividness in social media enhance users' beliefs about the attributes of virtual products (Fiore et al., 2005), further driving their reliance on the metaverse.

High-quality interactivity has been identified as a crucial factor in technology acceptance within metaverse environments (Xi et al., 2024). For example, Davis et al. (2009) highlight that interactivity in the metaverse includes users' ability to engage with virtual content in real time, making interactions between users more authentic and credible. In metaverse dating scenarios, the quality of interactions—such as engagement, satisfaction, safety, and compatibility—is also considered a key determinant of user attitudes.

Additionally, the high interactivity in virtual spaces enhances users' social presence and place attachment (Oleksy et al., 2023). This indicates that younger users, such as Generation Z, are more inclined to establish psychological belonging and dependency in virtual environments (Anto et al., 2024; Orazi et al., 2023). The attractiveness of visual elements is a crucial factor influencing users' attitudes and behaviors. Research shows that visual information is more likely to be remembered by users over the long term compared to text or numerical data (Ghapanchi et al., 2020). The subjective perception of the aesthetic quality of an information system, known as Perceived Visual Attractiveness (PVA), has been widely studied across various contexts, with its effects reflected in user loyalty (Cyr et al., 2006) and satisfaction (Chi, 2018). Previous studies have established a significant link between visual attractiveness and user attitudes. Furthermore, in mobile application contexts, visual attractiveness has been found to significantly influence users' perceived value and satisfaction (Liao et al., 2019). These findings provide a theoretical foundation for examining the relationship between visual attractiveness in the metaverse and users' attitudes toward escaping reality.

In the metaverse, visual attractiveness is exemplified through the design of avatar features, such as hairstyles, facial textures, skin tones, and hair colors. These features can significantly boost users' confidence in self-expression and create a positive first impression (Liao et al., 2019). Metaverse platforms further enhance users' visual experience by offering engaging interactive and immersive features. For university students, in particular, such visual experiences create memorable immersion and enjoyment, motivating their desire to escape reality.

The design of social scenarios also plays a critical role in the metaverse. Attractive virtual spaces, such as lounges, parks, beaches, and clubs, foster social activities and provide university students with opportunities to escape real-life challenges. The fun and interactivity of these designs are considered key drivers of user immersion in the metaverse (Dabholkar and Bagozzi, 2002; Richard et al., 2010). Against this backdrop, the metaverse not only enhances user engagement through highly personalized visual experiences but also evokes excitement and enthusiasm, fostering a more positive attitude among university students toward the metaverse.

Thus, we hypothesize: H5. The amount of time spent in the metaverse positively impacts university students' attitudes toward escaping to the metaverse.

H6. Interactivity has a positive impact on university students' attitudes toward escaping to the metaverse.
H7. Visual attractiveness positively influences university students' attitudes toward escaping to the metaverse.

To enhance conceptual clarity and address measurement transparency, the definitions and operationalizations of all key constructs used in this study are summarized in Appendix A. The table outlines the theoretical basis, definitions, and measurement sources of each variable, drawing on established frameworks such as Escape Theory, Self-Determination Theory, and the Elaboration Likelihood Model. To enhance conceptual clarity and measurement transparency, the definitions and operationalizations of all key constructs used in this study are summarized in Appendix A. The table outlines the theoretical basis, definitions, and measurement sources of each variable, drawing on Escape Theory, Self-Determination Theory (SDT), and the Elaboration Likelihood Model (ELM).

While SDT and Escape Theory originate from different theoretical traditions, they are compatible when understood as a sequential process. SDT proposes that the frustration of basic psychological needs—autonomy, competence, and relatedness—elicits negative affective states such as anxiety, helplessness, and alienation (Ryan and Deci, 2000). These emotional states align with the notion of psychological strain central to Escape Theory, which suggests that individuals experiencing such distress seek to escape from self-awareness or reality through alternative environments (Baumeister, 1991).

Accordingly, this study does not use SDT constructs as mere descriptive labels of student difficulties. Instead, they are conceptualized as theoretically grounded antecedents of psychological strain. In our model, need frustration leads to distress, which then motivates escapist behavior such as immersive engagement in the metaverse. This sequential framework allows SDT to explain the origin of discomfort and Escape Theory to explain the behavioral coping mechanism. Integrating both perspectives provides a unified explanation of how psychological vulnerability translates into digital escapism.

This Appendix provides readers with a concise reference for understanding how each construct was conceptualized and empirically applied in the study. Accordingly, this study distinguishes escapism predictors into two categories: internal psychological frustrations arising from the real world, and external technological affordances provided by the metaverse environment. This classification reflects the dual-path model of user motivation in immersive systems.

**Figure 1:**
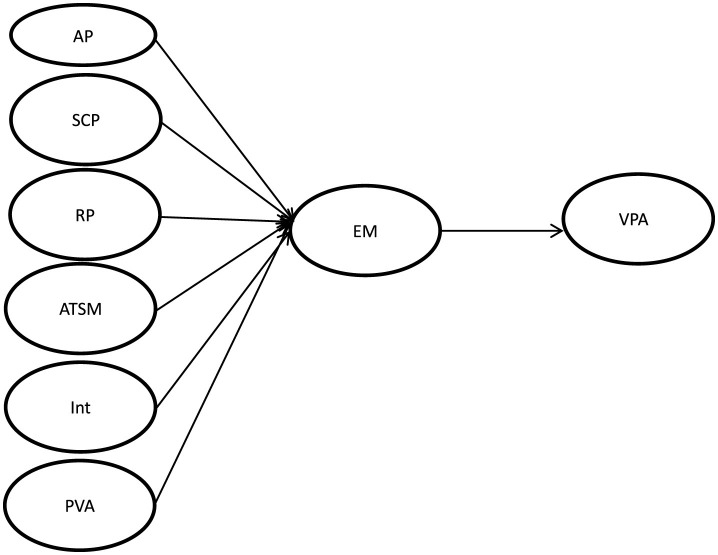
Research model.

Figure 1 reflects the integration of two theoretical perspectives: Escape Theory, which explains the motivational basis for digital detachment due to unmet psychological needs; and the Elaboration Likelihood Model, which distinguishes between internal factors processed via central routes and external environmental cues processed via peripheral routes. This mapping supports the structural equation model design and hypothesis development.

## 3 Data analysis

### 3.1 Data collection

This study takes Chinese college students as the research subjects. Between March and May 2024, questionnaires were distributed to students from eight universities in Mainland China, targeting individuals who have actively engaged in at least one metaverse platform, including Genshin Impact, Minecraft, Eggy Party, and Mini World. A total of 800 questionnaires were distributed, and after sampling, 585 valid questionnaires were collected, yielding an effective response rate of 83.6%.

Participants were required to have prior experience with one or more of the specified metaverse platforms to ensure the relevance of their responses to the research context. The sample covered students from diverse academic disciplines, year groups, and geographical regions, offering a broader representation of the Chinese college student population. Data collection adhered to strict ethical research guidelines, including informing participants about the purpose of the study, ensuring their participation was voluntary, and maintaining the confidentiality of their responses.

To test the research model, a survey instrument was developed with each construct measured using multiple items. Most items were adapted from existing measures in the related literature with confirmed content validity and reliability, and then modified to fit our research context.

Measurement Sources Virtual Place Attachment was measured using items adapted from Williams and Vaske (2003). Escaping to the Metaverse was assessed with items based on the scale developed and validated by Orazi et al. (2023). Amount of Time Spent in the Metaverse was measured by directly asking participants how many hours, on average, they spent on Metaverse during the past week. Interactivity was evaluated using items adapted from Jin et al. (2017). Perceived Visual Attractiveness was measured using items based on the guidelines provided by Venkatesh et al. (2013). Autonomy Problem, Study Competence Problem, and Relatedness Problem were assessed using items adapted from the framework developed by Chen et al. (2015), which focuses on basic psychological need satisfaction, frustration, and strength across cultures. All items were measured on a five-point Likert scale (1 = totally disagree; 5 = totally agree).

Because data were collected in China, translation and back-translation were adopted to ensure the translation quality. First, we consulted three professors of linguistics to understand the significance and readability of each item. The English questionnaire was then translated into Chinese with their help. Second, the Chinese questionnaire was translated into English by two PhD candidates otherwise unconnected with this study. Third, we compared the translated items with the original items in English. To ensure the consistency of the two English versions, we improved the translation and eliminated all inconsistencies.

A total of 700 questionnaires were distributed in five Chinese universities. Questionnaires were distributed and collected in June and July 2024, and a total of 585 questionnaires were received. Of these, 115 questionnaires were eliminated due to questions that were not answered, so the valid questionnaires numbered 585, resulting in a valid response rate of 83.6%. Male students accounted for 48.2% of respondents and female students for 51.8%; freshmen accounted for 25.1%, sophomores for 23.1%, juniors for 23.2%, and senior for 28.2%; students majoring in social science accounted for 57.4%, and students majoring in natural science for 42.6%; students from public universities accounted for 65.4%, and students from private universities for 34.6%.

To address the potential for common method bias (CMB) arising from the self-reported, single-source survey design, we conducted Harman's single-factor test and full collinearity assessments. The Harman's test revealed that the first factor accounted for 28.7% of the total variance, well below the 50% threshold (Podsakoff et al., 2003). Additionally, variance inflation factor (VIF) values for all constructs were below 3.3, indicating no substantial multicollinearity or CMB (Kock, 2015). These results suggest that CMB does not significantly threaten the validity of our findings.

## 4 Results and analysis

### 4.1 Evaluation of measurement model

Two-stage analysis was adopted for the data analysis. The reliability and validity of the measurement model were evaluated in the first stage, and the structural model was tested in the second stage to examine the research hypothesis (Black and Babin, 2019). The latent variable structural equation model (SEM) of Smart PLS 4.0 and SPSS 25 was adopted as the analytical method in this study. Table 1 shows that the composite reliability (CR) of each construct in this study ranges from 0.904 to 0.939, and the Cronbach's alpha (α) is greater than 0.700, indicating a high reliability for the constructs in this study. The square root of average variance extracted (AVE) of each dimension is greater than the correlation coefficient between the dimensions (Chin, 1998), which indicates a good discriminant validity for the constructs in the study. Correlation coefficients were all less than the square root of the AVE within one dimension, suggesting that each dimension in this study had good discriminant validity.

**Table 1:** Construct correlation coefficient matrix.

**Constructs**	**1**	**2**	**3**	**4**	**5**	**6**	**7**	**8**	**CR**	**AVE**
1. Interactivity (Int)	**0.838**								0.904	0.702
2. Relatedness problem (RP)	0.462	**0.891**							0.939	0.794
3. Perceived visual attractiveness (PVA)	0.530	0.472	**0.891**						0.939	0.794
4. Study competence problem (SCP)	0.635	0.430	0.436	**0.874**					0.929	0.765
5. Autonomy problem (AP)	0.531	0.373	0.466	0.535	**0.856**				0.916	0.733
6. Virtual place attachment (VPA)	0.524	0.394	0.525	0.462	0.417	**0.878**			0.931	0.772
7. Escaping to the metaverse (EM)	0.612	0.481	0.623	0.549	0.497	0.583	**0.876**		0.930	0.768
8. Amount of time spent in metaverse (ATSM)	0.563	0.456	0.508	0.494	0.515	0.467	0.586	**0.839**	0.905	0.704

The diagonal is calculated by the square root of the average variance extracted (AVE) of each dimension.

CR, composite reliability; AVE, average variance extracted. The bold values on the diagonal represent the square root of the Average Variance Extracted (AVE) for each construct.

Henseler et al. (2016) proposed that the heterotrait-monotrait ratio (HTMT) of correlations based on the multitrait-multimethod matrix can be used as a method to determine the discriminant validity. Table 2 shows that the HTMT values among constructs are all lower than 0.9, which also demonstrates that the constructs in this study are provided with good discriminant validity. According to the above analysis, this study has good construct validity.

**Table 2:** Heterotrait-monotrait (HTMT).

**Constructs**	**1**	**2**	**3**	**4**	**5**	**6**	**7**	**8**
1. Interactivity (Int)								
2. Relatedness problem (RP)	0.520							
3. Perceived visual attractiveness (PVA)	0.597	0.515						
4. Study competence problem (SCP)	0.723	0.474	0.478					
5. Autonomy problem (AP)	0.610	0.415	0.518	0.600				
6. Virtual place attachment (VPA)	0.597	0.431	0.576	0.513	0.468			
7. Escaping to the metaverse (EM)	0.696	0.531	0.686	0.611	0.558	0.645		
8. Amount of time spent in metaverse (ATSM)	0.655	0.513	0.571	0.563	0.591	0.527	0.665	

### 4.2 Data analysis and results

Smart PLS 3.0 was adopted for the structural model analysis in this study. The value of the standardized root mean square residual (SRMR) can be applied to evaluate the fit of the research model, which is between 0 and 1: the closer it is to 0, the better the fit is. However, the saturated model of the SRMR assumes that the number of paths in the structural model is the same as the number of related constructs in the measurement model, and the estimated model is calculated in terms of the sample dataset itself and the rows. When the SRMR of the saturated model and the estimated model is less than 0.08, it indicates a good fit for the model (Hu and Bentler, 1999). The value of the normed fit index (NFI) is between 0 and 1, where the closer it is to 1, the better the fit is, and a value of NFI greater than 0.8 means an acceptable fit.

According to the results calculated by Smart PLS, the value of the SRMR for the saturated model in this study is 0.043, and the value of the SRMR for the estimated model is 0.062, both of which are less than 0.080. The value of NFI is 0.872, which meets the requirements for fit. There is thus a good model fit for this study.

**Figure 2:**
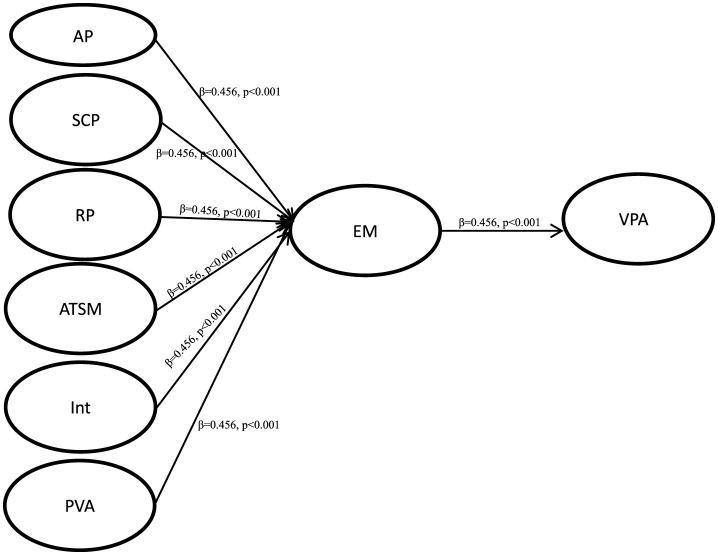
SEM analysis of the research model. *p < 0.05; **p < 0.01; ***p < 0.001.

After the evaluation and measurement results were found to be satisfactory, we evaluated the structural model, and examined the hypothesis through the percentage of variance and the significance of structural path. Figure 2 shows the test results of the PLS analysis containing control variables. Escaping to the Metaverse (EM) is significantly predicted by Autonomy problem (AP) (β = 0.456, *p* < 0.001), Study competence problem (SCP) (β = 0.525, *p* < 0.001) and Relatedness problem (RP) (β = 0.392, *p* < 0.001), thus supporting H1, H2 and H3. Amount of time spent in metaverse (ATSM) (β = 0.253, *p* < 0.01) and Interactivity (Int) (β = 0.324, *p* < 0.001) had a significant positive correlation with Escaping to the Metaverse (EM), thus supporting H4 and H5. Escaping to the Metaverse (EM) (β = −0.239, *p* < 0.001) was positively correlated with *i* Virtual place attachment (VPA), thus supporting H7. But Perceived visual attractiveness (PVA) (β = 0.018, p = 0.124) is not significant for Escaping to the Metaverse (EM), so H6 is not supported.

## 5 Discussions and conclusions

In this study, we proposed two objectives: to understand the motivations behind university students' escape to the metaverse and their subsequent attachment, as well as to examine the impact of specific internal and external factors on escapism. Accordingly, we categorized escapism motivations into two types: internal factors, including autonomy problems, competence issues, and relatedness problems; and external factors, including time spent in the metaverse, interactivity, and perceived visual attractiveness.

While we hypothesized all the proposed factors to be significant, we observed that H6 (perceived visual attractiveness) was not supported. This indicates that perceived visual attractiveness does not positively influence university students' intention to escape to the metaverse. This result contrasts with prior literature, which often emphasizes visual attractiveness as a critical factor for engaging users in virtual worlds and digital platforms. For example, Trunfio and Rossi (2022) found that enhanced visual elements significantly improved user immersion, leading to longer time spent in virtual worlds. Similarly, Lee et al. (2019) identified visual attractiveness as a primary motivator for users' initial entry into virtual reality environments.

However, our data suggest that while visual attractiveness may enhance the appeal of the metaverse experience, it is not a primary driver for university students' choice to escape reality and enter the metaverse. This could be attributed to the more complex and multifaceted motivations for engaging with the metaverse, which extend beyond the scope of visual appeal. Factors such as real-world stress, the need for escapism, social connections, and the pursuit of immersive and interactive experiences likely play more critical roles in students' decisions to engage with the metaverse (Pal and Arpnikanondt, 2024). This aligns with Edwards and Larson (2020) argument that while visual attractiveness may initially draw users in, long-term retention is determined by content depth, social interaction, and the ability to meet psychological needs.

Additionally, the diversity and complexity of the metaverse may result in varying impacts of visual attractiveness on different user groups. For instance, some users may prioritize interactivity and social opportunities over visual appeal. Among university students, in particular, social interaction and identity construction may outweigh the importance of visual elements (Wang et al., 2024). This differentiated impact highlights a future research direction: exploring how perceptions of visual attractiveness vary across user groups and how these differences influence behavior. Future studies could segment audiences by age, gender, or social needs to further uncover the mechanisms of visual attractiveness in different contexts (Mansoor et al., 2024).

Although escapism among university students has been discussed in the context of online gaming or social media networks, it remains an underexplored area in the metaverse (Han et al., 2022). Thus, while our results cannot be directly compared to other metaverse-related studies, prior research on escapism in gaming or social media has identified drivers such as enjoyment (Lee et al., 2021; Warmelink et al., 2009) and anticipated time investment (Coyne et al., 2020). To understand potential outcomes, it is essential to examine the factors driving escapism. Enjoyment, immersion, and even personality traits may influence escapism behaviors (Arpaci et al., 2022; Han et al., 2022; Zeuge, 2020). In contrast, our study introduces a novel predictive framework for escapism based on motivations driven by internal and external factors. The motivation-based approach adopts an empirical perspective, focusing on negating certain aspects of life, such as real-life challenges and frustrations (Warmelink et al., 2009; Zeuge, 2020).

As the final outcome variable, much of the existing metaverse research examines factors like usage intention, social sustainability, or user behavior (Akour et al., 2022; Arpaci et al., 2022; Gupta et al., 2024). Given the pace of technological advancement, university students seamlessly navigate between “real” and “virtual” worlds. Thus, considering attachment to virtual spaces as a critical indicator of metaverse adoption is logical. We observed that students who tend to spend more time in the metaverse score higher in virtual place attachment. Consequently, immersive time should be closely linked with attachment to and drivers of virtual spaces.

Overall, our findings align with prior empirical research confirming that students facing autonomy, competence, or social pressures are more inclined toward digital escape and virtual place attachment (Orazi et al., 2023; Pal and Arpnikanondt, 2024). By demonstrating its applicability to students experiencing autonomy problems, competence issues, and relatedness problems, and showing that time spent in the metaverse and quality interactivity increase the willingness to escape to the metaverse, we provide novel insights into this area.

### 5.1 Theoretical implications

This study makes several theoretical contributions. First, it extends both Escape Theory and the Elaboration Likelihood Model (ELM) into immersive virtual environments. Specifically, we refine Escape Theory by unpacking its antecedents into discrete psychological frustrations—autonomy, competence, and relatedness—grounded in Self-Determination Theory. This granularity advances the theory's explanatory power in digitally mediated contexts.

Second, unlike prior studies that treat escapism as a monolithic response, our results reveal distinct cognitive pathways—central (need-based) and peripheral (design-based)—shaping escapist intention and virtual attachment.

Finally, by mapping both internal states and environmental cues into a single explanatory framework, this study proposes a hybrid psychological-technical model of escapism, advancing theoretical discourse on how technology mediates user behavior beyond instrumental use.

Moreover, the insignificant result of H6 challenges the conventional emphasis on visual attractiveness as a primary factor in sustaining user engagement. Our findings suggest that in complex virtual environments like the metaverse, visual aesthetics alone may not drive long-term escapism. Instead, factors such as interactivity, emotional connection, and autonomy emerge as more influential. This echoes Makransky and Lilleholt (2018) view that meaningful virtual experiences require integration of multisensory and psychological dimensions.

In conclusion, this study provides a new perspective on the theoretical understanding of user behavior in the metaverse and suggests that future research should place greater emphasis on the multidimensional nature of user experiences. This multidimensional approach is of significant importance to academic research.

### 5.2 Practical significance

This study offers concrete, domain-specific insights for educational administrators, mental health professionals, and metaverse developers seeking to address the psychological antecedents of digital escapism among university students.

For educational institutions, our findings highlight that autonomy frustration, competence challenges, and lack of relatedness are key psychological drivers of students' metaverse escapism. To mitigate these factors, universities should enhance autonomy-supportive learning environments (e.g., self-directed modules, flexible deadlines), strengthen academic feedback systems to build perceived competence, and promote inclusive communities that foster social connectedness. These interventions may reduce students' psychological strain and reliance on virtual escape.

For metaverse developers, the results underscore the importance of moving beyond visual appeal to designing systems that support users' psychological wellbeing. Developers should implement personalized recommendation algorithms that respond to emotional states, encourage adaptive goal setting, and promote positive virtual routines. With emerging brain-computer interface (BCI) technologies, platforms may even detect affective cues in real time and redirect users toward emotionally restorative content or supportive communities.

Importantly, the study reveals the dual nature of escapism. While temporary digital withdrawal may relieve negative emotions and enhance short-term resilience, repeated avoidance can foster maladaptive behavior patterns. To address this, we propose a responsible use framework, including a customizable warning system that monitors excessive usage among university student users. This system could issue context-sensitive reminders (e.g., during prolonged entertainment sessions) and be guided by an interdisciplinary committee comprising educators, developers, and mental health professionals.

By synthesizing insights across digital escapism and metaverse psychology, this study advances an integrative framework that links psychological strain with virtual engagement. Our model responds to recent empirical findings while offering a unified lens to interpret user attachment in immersive environments.

### 5.3 Research limitations

In this study, we argue that escapism in the metaverse is a key driving force behind individuals' attachment to this virtual space. Our findings provide a foundational framework for explaining how different factors drive university students to escape to the metaverse and develop attachment to this virtual place. These insights offer valuable guidance to metaverse developers, helping them devise strategies to attract more young users to the platform and foster connections, thereby showcasing their influence.

While this study addresses the gap in understanding the drivers of university students' escapism to the metaverse and challenges some assumptions previously held by metaverse stakeholders, several limitations remain, which may affect the generalizability and breadth of the findings.

First, the sample in this study was limited to university students from a specific region and age group, which may restrict the generalizability of the results. Individuals from different cultural backgrounds, age groups, or educational contexts may exhibit varying behavioral patterns and motivations. Therefore, the findings may not be directly applicable to other groups or regions. Future research could expand the sample to include a broader audience, validating whether the results are universally applicable.

Second, this study employed a cross-sectional research design, with data collected at a single point in time. Such a design limits the ability to infer causality, particularly regarding the relationship between real-world challenges and escapism behavior in the metaverse. Future research could adopt a longitudinal design to better capture the dynamic relationships between these variables.

Third, the conceptualization and definition of the “metaverse” used in this study may become outdated as technology evolves. Metaverse technologies and user experiences are rapidly evolving, with different platforms and applications potentially offering varied user experiences and motivational factors. Future studies should continuously monitor technological advancements and reassess related behavioral patterns and theoretical frameworks within new technological contexts.

Finally, although this study explored several factors influencing university students' escapism to the metaverse, other potential variables were not included. Factors such as individual mental health conditions, social media usage habits, and familiarity with virtual reality technology could also influence user behavior. Future research could investigate these variables to develop a more comprehensive understanding of the factors affecting metaverse usage behavior.

The limitations of this study highlight directions for future research to address existing gaps and further enrich the understanding of user behavior in the metaverse.

## Data Availability

The raw data supporting the conclusions of this article will be made available by the authors, without undue reservation.

## Appendix

**Appendix A:** Definitions and measurement sources of key constructs.

**Variable**	**Source theory**	**Role in model**	**ELM processing route**
Autonomy problem	Self-determination → escape theory	Psychological antecedent	Central route (high relevance)
Study competence problem	Escape theory	Psychological antecedent	Central route
Relatedness problem	Escape theory	Psychological antecedent	Central route
Time spent in metaverse	Media affordance + ELM	Environmental enabler	Peripheral route
Interactivity	ELM (cue) + immersion theory	Technological facilitator	Peripheral route
Visual attractiveness	ELM (aesthetic cue)	Sensory stimulus	Peripheral route
Escapist intention	Escape theory outcome	Mediator	N/A
Virtual place attachment	Consequence of escape	Final outcome	N/A
